# Beneficial effects of paeoniflorin on non-alcoholic fatty liver disease induced by high-fat diet in rats

**DOI:** 10.1038/srep44819

**Published:** 2017-03-16

**Authors:** Zhihong Ma, Li Chu, Hongying Liu, Weijie Wang, Jieru Li, Wenzao Yao, Jianfeng Yi, Yue Gao

**Affiliations:** 1 Yichun University, Key Laboratory for Research on Active Ingredients in Natural Medicine of Jiangxi Province, Yichun, 336000, China; 2 Hebei University of Chinese Medicine, Department of Immunology and Pathobiology, Shijiazhuang, 050200, China; 3 Hebei Medical University, Department of Pharmacology, Shijiazhuang, 050017, China; 4 Hebei General Hospital, Department of Infectious Diseases, Shijiazhuang, 050051, China; 5 The Second Hospital of Hebei Medical University, Department of Surgery, Shijiazhuang, 050000, China; 6 Hebei University of Chinese Medicine, Department of Physiology, Shijiazhuang, 050200, China; 7 Beijing Institute of Radiation Medicine, Department of Pharmacology and Toxicology, Beijing, 100850, China

## Abstract

Non-alcoholic fatty liver disease (NAFLD) is the most prevalent form of chronic liver diseases. This study sought to evaluate the insulin-sensitizing effect of paeoniflorin (PF) on high-fat diet-induced NAFLD and possible molecular mechanisms. Male Sprague Dawley rats were fed a high-fat diet (HFD) for 10 weeks to establish the NAFLD model, and PF (20 mg/kg/d) was gavaged to the NAFLD rats for another four weeks. Our results demonstrated that HFD resulted in hepatocellular ballooning, micro-/macrovesicular steatosis, and oxidative stress in the liver, accompanied by increased serum total cholesterol (TC), triglyceride (TG), free fatty acid (FFA), alanine aminotransferase (ALT), and aspartate aminotransferase (AST) levels and homeostasis model of insulin resistance (HOMA-IR) index. PF treatment improved the biochemical and histopathological changes in NAFLD rats. Moreover, we also found that PF could inhibit lipid ectopic deposition via regulating lipid metabolism (inhibiting lipid synthesis of cholesterol and *de novo* pathway), and exert insulin sensitizing effect by regulating the insulin signaling pathway IRS/Akt/GSK3β and anti-oxidation. The study findings suggest that PF has therapeutic potential against NAFLD and that it acts through multiple signaling pathways.

Non-alcoholic fatty liver disease (NAFLD) is regarded as the manifestation of metabolic syndrome in the liver^1^, and has been a major public-health concern in the world, affecting more than 40% of population in some countries^2^,^3^. NAFLD includes simple steatosis, steatohepatitis (NASH), fibrosis, and cirrhosis. About 10%～29% of patients with NASH will further transition to cirrhosis within a 10-year period^4^. Moreover, recent data suggest that NAFLD and NASH are linked to increased cardiovascular risks^5^. Regretfully, there is no available pharmacological treatment for NAFLD^6^, thus, effective drugs or compounds for treating patients with NAFLD are urgently required.

Paeoniflorin (PF) (Fig. 1), a major pharmacologically active component from roots of peonies, has been reported to possess anti-inflammatory^7^, antioxidant^8^, antihyperlipidemic activities^9^ and hepatoprotection^10^, with low toxicity and few side effects^11^,^12^. Recent studies indicated that PF improved insulin resistance (IR) in NAFLD animal models by promoting fatty acid oxidation through the activation of peroxisome proliferator-activated receptor-alpha (PPARα)^13^ and by increasing the activity of AMP-activated protein kinase (AMPK)^14^. Our previous research demonstrated that PF had anti-inflammatory and hepatoprotective effects in NASH rats and that these effects due to inhibition of the Rho kinase/NF-κB signaling pathway in liver with NASH^11^.

It is widely accepted that the hepatic lipid accumulation of NAFLD represents hepatic manifestations of impaired systemic insulin network^15^. One cause of IR in NAFLD is mediated by impaired insulin signaling pathway, for example, impairment of the insulin receptor substrate (IRS)-1/Akt/glycogen synthase kinase (GSK) 3β pathway^6^. Serine/threonine phosphorylation of IRS is associated with IR, and it is enhanced by elevated reactive oxygen species (ROS) levels and/or multiple inflammatory cytokines such as tumor necrosis factor (TNF) α^16^. Elevation of hepatic ROS levels has been proposed as a link between elevated serum free fatty acids (FFAs) and hepatic IR^17^. In this study, we hypothesized that PF could suppress hepatic oxidative stress and then restore the impaired IRS1/Akt/GSK3β signaling pathway, leading to an improvement in IR. We evaluated the insulin-sensitizing effects of PF in a NAFLD rat model and explored its possible therapeutic mechanisms.

## Results

### Effects of PF on body weight and liver index

**Figure 2:**
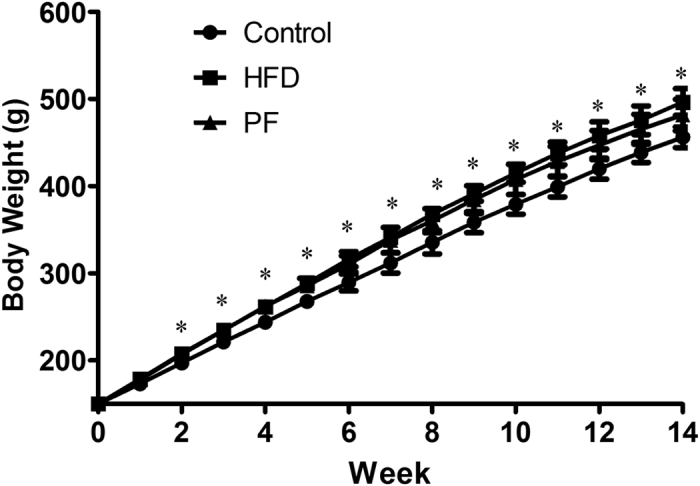
Effects of PF on trends of body weight. Values are means ± SD. n = 8 per group. *P < 0.05 vs. Control group.

Rats fed with HFD lasting fourteen weeks had a marked increase in final body weight and in the liver index (liver wet weight/body weight × 100%) compared with the Control rats (P < 0.05) (Table 1). Treatment with PF (20 mg/kg/d), the final body weight tended to be lower compared with the rats in the HFD group, although it had no statistics difference (P > 0.05) (Fig. 2). Additionally, there was no significant difference in food intake among the three groups (data not shown). However, PF treatment dramatically decreased the liver index compared with the Control rats (P < 0.05) (Table 1).

### Effects of PF on serum and hepatic lipid profiles

**Figure 3:**
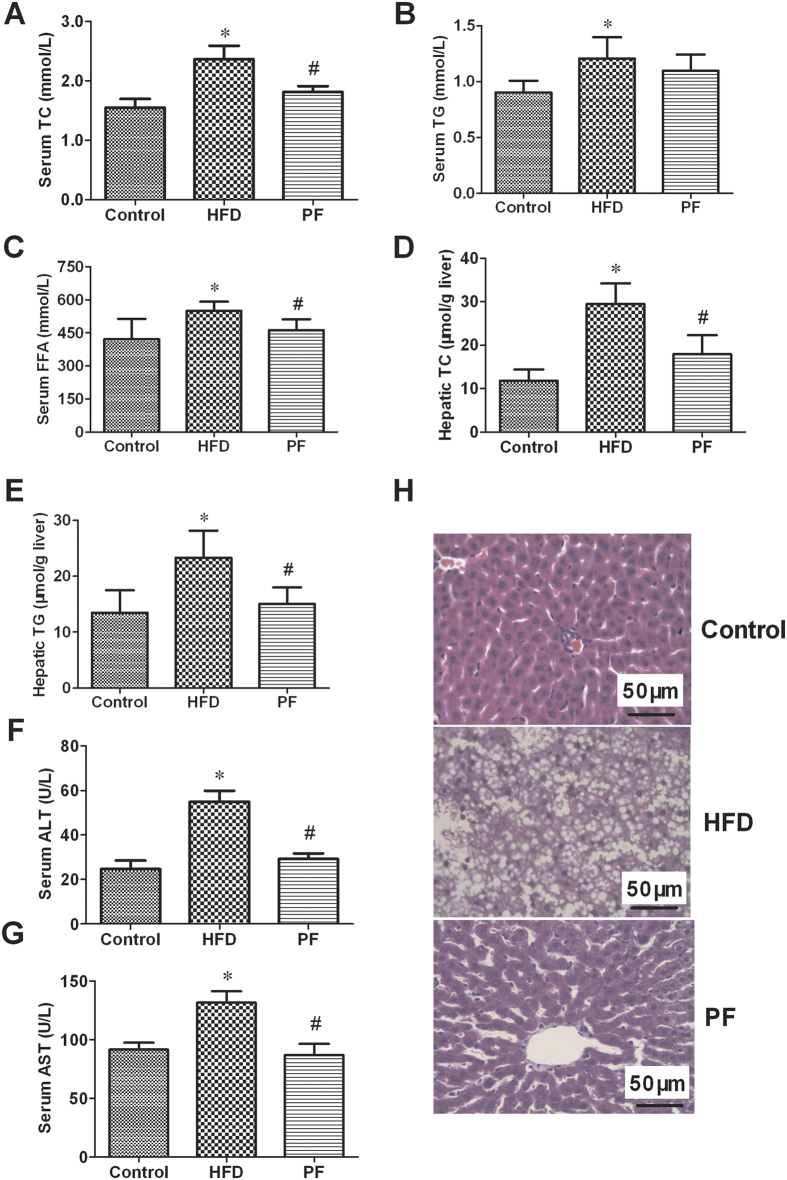
Effects of PF on lipid profiles and liver function. Serum TC (**A**), TG (**B**), FFA (**C**), hepatic TC (**D**) /TG (**E**), serum ALT (**F**) /AST (**G**) levels, and representative microscopic photographs of liver sections stained with hematoxylin–eosin (Magnification, 400×) after treatment with physiological saline (HFD) or paeoniflorin (**H**). Values are means ± SD. *P < 0.05 vs. Control group; ^#^P < 0.05 vs. HFD group. n = 8 per group.

As shown in Fig. 2, the serum levels of TC (Fig. 3A), TG (Fig. 3B), and FFA (Fig. 3C) of the HFD group were higher than those of the Control rats (all P < 0.05). However, PF treatment reduced the serum TC and FFA levels (both P < 0.05), but not the serum TG level, when compared with the levels observed in the HFD group.

In line with the changes of serum lipids, hepatic TC (Fig. 3D) and TG (Fig. 3E) levels were significantly higher in the HFD rats compared with the Control rats (P < 0.05), and these were both decreased by PF treatment.

### Effects of PF on liver injury

ALT and AST are two hepatic-specific enzymes that reflect the degree of early acute liver damage. As shown in Fig. 2, the serum ALT (Fig. 3F) and AST (Fig. 3G) activities in the HFD rats were higher than those of the Control rats (both P < 0.05). PF treatment markedly lowered the serum ALT and AST activities compared to those of the HFD rats (both P < 0.05).

Histological analysis of HE staining (Fig. 3H) indicated that the livers of the HFD-fed rats showed significant lipid droplet accumulation, acinar and portal inflammation, infiltration of macrophages and lymphocytes. PF treatment significantly improved the hepatic histology induced by HFD.

### Effects of PF on the expression of lipid metabolic regulators in the liver

**Figure 4:**
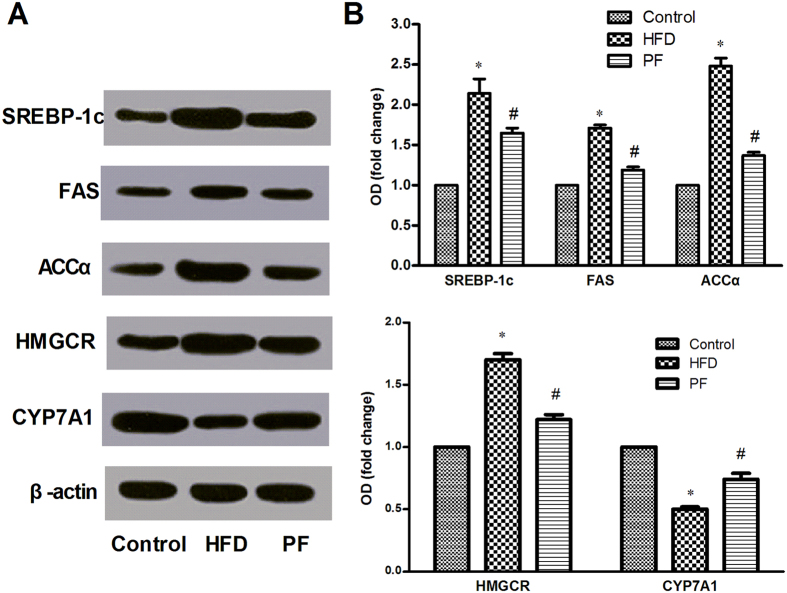
Effects of PF on the expression of lipid metabolic regulators in the liver. The protein expression of hepatic SREBP-1c, FAS, ACCα, HMGCR and CYP7A1 to control β-actin were determined by western blotting after treatment with physiological saline (HFD) or paeoniflorin. (**A**) Representative Western blotting results. (**B**) Densitometric analysis of immunoblot data. Values are means ± SD. *P < 0.05 vs. Control group; ^#^P < 0.05 vs. HFD group. n = 8 per group.

To further elucidate the mechanism why PF inhibits excessive deposition in liver, we detected several enzymes affecting lipid synthesis in the liver tissue by western blotting. As shown in Fig. 4, several lipid synthesis-associated enzymes changed. In the way of *de novo* synthesis, protein expressions of sterol regulatory element binding protein (SREBP)-1c, fatty acid synthase (FAS) and acetyl-Coenzyme A carboxylase (ACCα) were higher in HFD rats than in Control rats. But these changes were reversed by PF treatment (all P < 0.05). In the way of cholesterol metabolism, the higher expression of HMG-CoA reductase (HMGCR) and lower expression of cholesterol 7-alpha-hydroxylase (CYP7A1) were seen in HFD rats compared with the Control rats (both P < 0.05). PF treatment could inhibit the expression of HMFCR, but upregulate the expression of CYP7A1 (both P < 0.05).

### Effects of PF on systemic and hepatic insulin resistance

**Figure 5:**
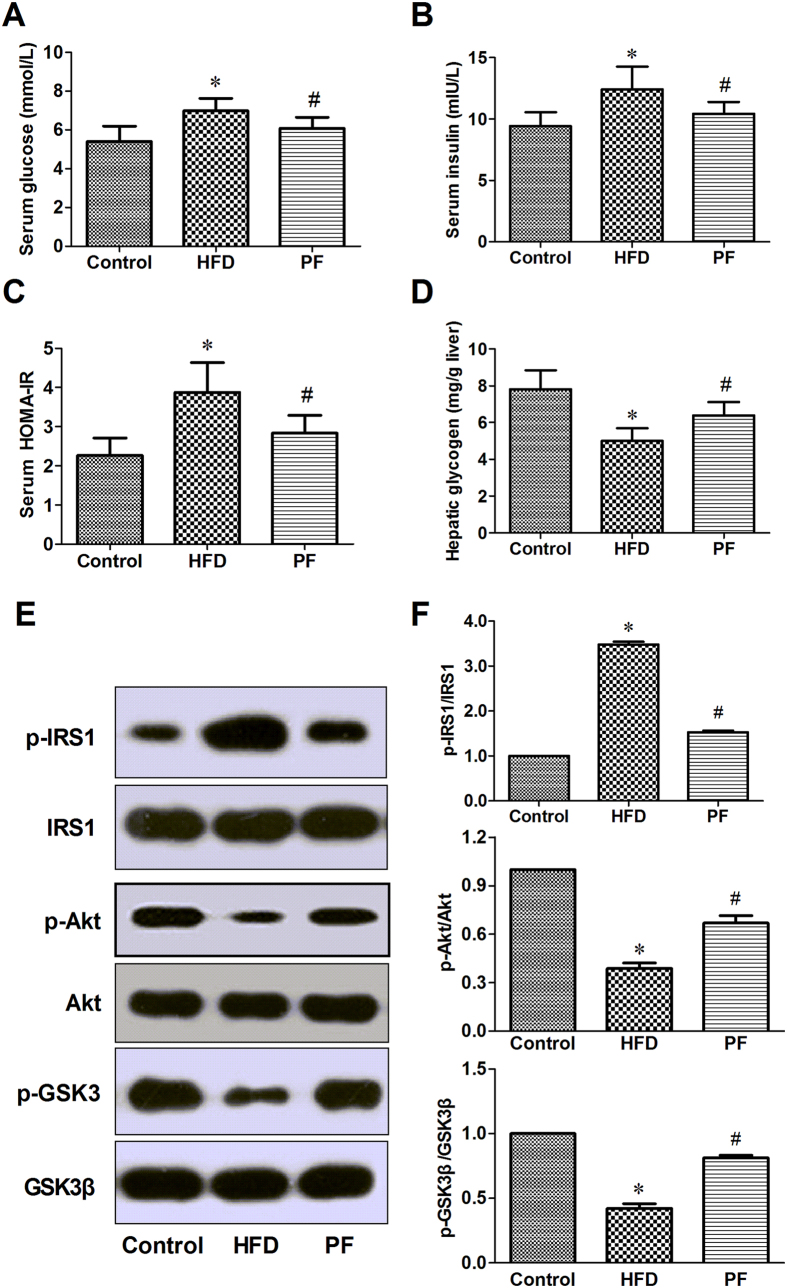
Effects of PF on insulin resistance and insulin signaling pathway. Serum fasting glucose (**A**), fasting insulin (**B**), HOMA-IR values (**C**), hepatic glycogen content (**D**) and western blotting results of IRS-1/Akt/GSK3β protein expression (E and F) after treatment with physiological saline (HFD) or paeoniflorin. Values are means ± SD. *P < 0.05 vs. Control group; ^#^P < 0.05 vs. HFD group. n = 8 per group.

Systemic IR was evaluated using the homeostasis model of insulin resistance (HOMA-IR) index, which is calculated as fasting serum glucose fasting × serum insulin/22.5. Lower HOMA-IR value indicates a higher degree of insulin sensitivity^18^. Intriguingly, rats fed the HFD had strikingly higher fasting blood glucose and insulin (Fig. 5A and B); furthermore, HOMA-IR values (Fig. 5C) were higher than those in the Control rats. PF treatment resulted in an almost complete normalization of these indexes (P < 0.05).

A decrease in hepatic glycogen content is a marker of hepatic IR. Similar to systemic IR, HFD rats showed significantly lower hepatic glycogen contents, compared with the Control rats (P < 0.05). After PF treatment, the rats exhibited a significant recovery in hepatic glycogen content compared with the HFD rats (P < 0.05) (Fig. 5D).

### Effects of PF on the hepatic insulin signaling pathway

To elucidate the potential mechanism underlying the observed PF-mediated improvement in IR, we evaluated intracellular insulin signaling pathway molecules in the liver tissue by western blotting. Representative western blot analyses of IRS-1, p-IRS-1 (Ser^307^), Akt, p-Akt (Ser^473^), GSK3β and p-GSK3β (Ser ^9^) are displayed in Fig. 4. The data clearly show that HFD-fed rats had impaired insulin signaling, as evidenced by increased serine phosphorylation of IRS-1 and decreased phosphorylation of Akt and GSK3β compared with Control rats. PF treatment resulted in a marked restoration in protein expression of these markers. No difference was observed in hepatic total IRS-1, Akt and GSK3β protein expression among the three groups (Fig. 5E and F).

### Effects of PF on serum and hepatic oxidative stress

**Figure 6:**
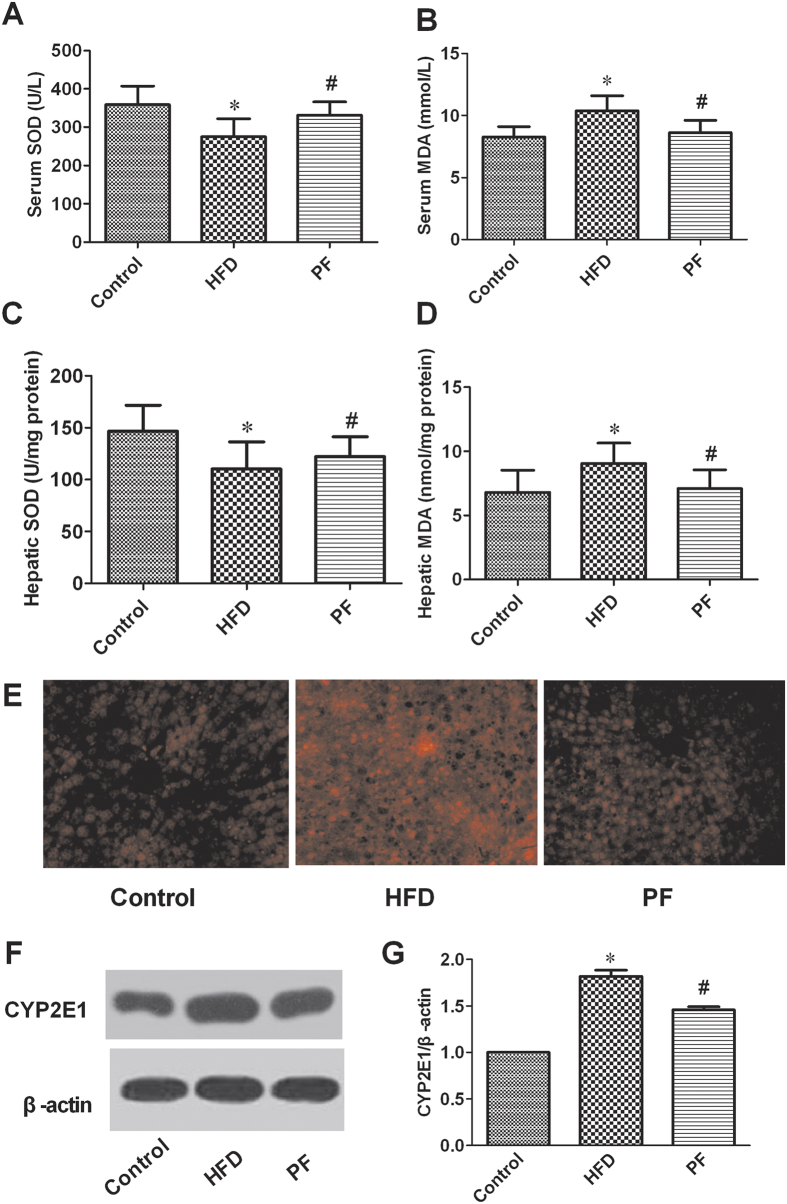
Effect of PF on oxidative stress. Serum SOD (**A**) /MDA (**B**), hepatic SOD (**C**)/MDA (**D**), ROS production detected with DHE staining (Magnification, 400×) (**E**) and CYP2E1 protein expression (**F** and **G**) after treatment with physiological saline (HFD) or paeoniflorin. Values are means ± SD. *P < 0.05 vs. Control group; ^#^P < 0.05 vs. HFD group. n = 8 per group.

As shown in Fig. 6, the HFD rats had lower SOD activities in serum and liver tissue than those of the Control rats (Fig. 6A and C). Rats treated with PF showed higher serum and hepatic SOD activities than those of HFD rats (both P < 0.05).

In contrast to SOD, the serum and hepatic MDA levels (Fig. 6B and D) in the HFD rats were significantly higher compared with those in the Control rats (both P < 0.05). Meanwhile, the PF treated groups showed significantly reduced MDA levels compared with the HFD rats (both P < 0.05).

ROS production in liver was detected by DHE staining. As shown in Fig. 6E, DHE fluorescence in the livers of the HFD rats was significantly elevated compared with the Control rats. PF treatment mitigated hepatic ROS production compared with the HFD rats.

### Effects of PF on CYP2E1 protein expression in liver

CYP2E1 has been suggested to mediate the ROS generation that induces oxidant stress in non-alcoholic fatty liver disease^19^,^20^. Western blot analysis (Fig. 6F and G) showed that the protein expression of CYP2E1 was increased in the livers of the HFD rats as compared with the livers of the Control rats; PF treatment could down-regulate CYP2E1 protein expression.

## Discussion

The aim of the present study was to investigate the effects of PF on HFD-induced NAFLD. In particular, we explored the effects of PF on improving fatty liver and IR in NAFLD rats based on its effects on the lipid metabolism-associated regulators and insulin-associated IRS/Akt/GSK3β pathway. Our data suggest that PF could exert beneficial effects on NAFLD, in part, through regulating lipid metabolism, inhibiting oxidative stress and by regulating the IRS/Akt/GSK3β pathway.

Animal models of NAFLD can be divided into two types: those caused by genetic mutations, and those induced by dietary or pharmacological modifications. Because caloric overconsumption is a major factor in NAFLD development in the clinical condition, recent studies have successfully established rat NAFLD model using HFD^21^. It was found that rats fed with a long-term HFD (standard rat chow supplemented with 2% cholesterol plus 15% lard) can serve as an experimental model of NAFLD, with typical hepatic lesions, including hepatomegaly, hepatocyte ballooning, steatosis and steatohepatitis. This liver injury is accompanied by increased HOMA-IR index and elevated levels of serum TC, TG, FFA, ALT and AST compared with the rats fed a standard chow diet^22^. These results suggested that HFD caused dyslipidemia, IR, hepatic steatosis, and impaired liver function, furthermore, suggested a successful animal model was established. However, treatment with PF significantly reduced the liver index and decreased the elevation of serum TC, FFA, ALT and AST levels. Moreover, pathological injuries were relieved following PF treatment, consistent with other reports^13^,^14^.

Our study also demonstrated that treatment with PF significantly reduced lipid accumulation in liver, which is consistent with our previous study^11^ and the report by Zhang *et al*.^14^ in animals. Therefore, to clarify the mechanism of PF involved in inhibiting lipid accumulation, we detected the several lipid metabolism-associated enzymes. SREBP-1c is an important transcription factor which impacts several genes involved in the *de novo* pathway, such as FAS and ACCα. Our study suggested that PF could suppress *de novo* lipid synthesis in the liver by suppressing the protein expression of SREBP-1c, FAS and ACCα, in consistent with Zhang’ report in which PF could suppress the gene expression of SREBP-1c, FAS and ACCα^14^. In the way of cholesterol metabolism, HMGCR and CYP7A1 are the limited enzymes in the synthesis process of cholesterol and bile acid synthesis, respectively. CYP7A1 converts cholesterol to 7-alpha-hydroxycholesterol. Our results found that PF could inhibit the protein expression of HMGCR and promote the protein expression of CYP7A1, which may result in a reduction of cholesterol in liver. Taken together, our results suggested that PF could regulate lipid synthesis and metabolism via multiple signaling pathways.

A number of clinical and animal studies have confirmed that IR is the first step and physiopathological key towards NAFLD development and its severity^6^. Insulin acts in the liver via multiple pathways; one is participated in the regulation of hepatic glycogen synthesis and includes insulin receptor β (IRβ), insulin receptor substrates (IRS), phosphatidylinositol 3-kinase (PI3K), protein kinase B (PKB/Akt), and glycogen synthase kinase-3β (GSK-3β). IRS1 is the major insulin receptor effector that contributes to the signaling that regulates glucose homeostasis in the liver, while phosphorylation of IRS-1 at Ser^307^ is considered a negative feedback marker that inhibits insulin signaling, leading to IR^23^,^24^. Elevations in serum FFA, in several inflammatory cytokines (for example TNF-α), and in ROS are important mediators of IR in obesity and NAFLD because they induce IR at the level of the IRS proteins^25^. The kinase Akt, also called protein kinase B, has been reported to mediate insulin-induced glycogen synthase activation and glucose uptake, and it is activated mainly at two regulatory sites, Thr 308 and Ser 473. Activated Akt, in turn, inhibits glycogen synthase kinase 3β (GSK3β) by phosphorylating it at Ser^9^. Inactivated GSK3β promotes glycogen synthesis. A mass of evidence suggests that deregulation of the phosphatidylinositol 3-kinase (PI3K)/AKT signaling pathway in hepatocytes is a common molecular mechanism involved in metabolic dysfunctions including obesity, metabolic syndrome, and NAFLD^26^.

The HOMA-IR index is used to assess systemic IR, and higher HOMA-IR values indicate a higher degree of IR. Conversely, decreased hepatic glycogen content is associated with hepatic IR. In our study, elevated HOMA index values that were associated with HFD administration were restored to normal control levels after PF treatment. Moreover, our data clearly showed that PF could inhibit phosphorylation of IRS1 at Ser^307^ and upregulate the phosphorylation of Akt and GSK3β. Likewise, PF significantly lowered the HFD-induced rise in FFA level, indicating that the decrement in FFA observed in the treatment groups might have contributed to the amelioration of IR and to the improvement in insulin signaling. This result is similar to Chen’s report, in which PF improved IR in HFD-induced rats by regulating lipid synthesis, including increasing phosphorylation of AMPK and by decreasing the gene expression of SREBP-1c and FAS^13^. In the current study, hepatic triglyceride levels were markedly lower in PF rats, and this result is consistent with the reduction in serum FFAs and the improvement in cellular insulin sensitivity observed in the PF rats.

Oxidative stress has been thought to play a key role in NAFLD^27^. NAFLD patients have both elavated production of reactive oxygen species (ROS) and lowered antioxidant capacity^28^. Elevated plasma FFAs are taken up by the liver, accelerating the rate of hepatic β-oxidation, which is the main resource of hepatic ROS production. In addition, IR can also trigger the release of ROS by upregulating microsomal lipid peroxidation and by downregulating mitochondrial β-oxidation. Lipid peroxidation further results in the generation of by-products, such as malondialdehyde (MDA), which could activate of the inflammatory response and, consequently, cause cellular damage^29^. In the present study, ROS production and the lipid peroxidation indicator malondialdehyde (MDA) were increased in both serum and liver tissue in the HFD rats, whereas the activities of SOD, a potent antioxidant, were decreased in both serum and liver tissue. Rats treated with PF exhibited decreased levels of ROS and MDA and had enhanced SOD activities in both serum and liver tissue. Our results support previous reports demonstrating that PF can inhibit lipid peroxidation in ANIT-induced cholestasis^8^ and in hepatic ischemia/reperfusion injury^10^. The potential effect of PF in preventing oxidative stress could be due to the ability to reduce free radical production or through increased free radical scavenging activity.

CYP2E1 is a major microsomal source of cellular ROS, and increased hepatic CYP2E1 protein expression and activity in association with enhanced ROS generation have recently been reported in animal models of obesity and in patients with obesity and nonalcoholic fatty liver disease^30^,^31^. In this study, increased hepatic CYP2E1 protein expression was seen in HFD rats, PF can inhibit the expression of CYP2E1 in liver. However, the effect of PF on the activity of CYP2E1 is unknown and requires further research.

In conclusion, the present results suggest that the protective effect of paeoniflorin in NAFLD treatment may result from insulin-sensitizing effects though the regulation of the IRS1/Akt/GSK3β pathway and from reduced ROS production through the suppression of CYP2E1. Paeoniflorin, therefore, may be considered as a potential therapeutic agent for NAFLD, but more in-depth studies need to fully elucidate its mechanisms of action and its clinical effects.

## Methods

### Ethics statement

All methods of this study were carried out in accordance with the 1996 National Institutes of Health Guide for the Care and use of Laboratory Animals. And all experimental protocols were approved by the Animal Ethics Committee of Hebei University of Chinese Medicine (approval number: HEBUCM-2015-03; approval date: March 01, 2015).

### Materials

PF (purity > 98%) was purchased from Zelang Medical Technology (Nanjing, China). The radioimmunoassay (RIA) kits for the determination of serum insulin (INS) levels were obtained from Northern Institute of Biotechnology (Beijing, China). Kits for determining serum total cholesterol (TC), triglycerides (TG), fasting blood glucose (FBG), free fatty acids (FFAs), aspartate aminotransferase (AST), alanine aminotransferase (ALT), superoxide dismutase (SOD), malondialdehyde (MDA) and hepatic glycogen content were obtained from Jian Cheng Biological Engineering Institute (Nanjing, China). The ROS Fluorescent Probe - dihydroethidium (DHE) was purchased from Nanjing KeyGen Biotech. Inc. (Nanjing, China). Primary antibodies of IRS-1, p-IRS-1 (Ser^307^), Akt, p-Akt (Ser^473^), GSK3β, p-GSK3β (Ser^9^), CYP2E1, β-actin and horseradish peroxidase-conjugated secondary antibodies, and the enhanced chemiluminescence (ECL) detection system were purchased from Bioworld Technology, Inc. (Minnesota, USA). Primary antibodies of SREBP-1c, FAS, ACCα, HMGCR and CYP7A1 were obtained from Biosynthesis Biotechnology (Beijing, China).

### Animal and experimental protocols

Twenty-four male Sprague-Dawley rats weighing 140–160 g were obtained from the Hebei Experimental Animal Center (Shijiazhuang, China). All rats were housed in cages under standard conditions with free access to water and to the diets corresponding to their assigned treatment group.

The rats were randomized to the following three groups (each group n = 8): normal control (Control), HFD group (HFD) and HFD plus PF 20 mg/kg (PF). The Control group rats were fed a standard diet, and the HFD and PF groups received the HFD, which was standard chow plus 2% cholesterol and 15% lard)^32^. Ten weeks later, the rats in the PF and HFD groups were gavaged with PF at a dose of 20 mg/kg/day^11^ or with an equal volume of distilled water, respectively.

Food intake was monitored daily and body weight was measured weekly during the experiment. Spilled food was collected and compensated in readjusting the calculation of food intake. After the additional four-week treatment, blood was collected through the femoral aorta, and the liver tissues were excised, weighed, and processed for subsequent analyses.

### Determination of serum biochemistry

Serum TG, TC, FFA, FBG, ALT, AST, SOD and MDA were determined with commercially available kits according to the instructions of the kits. The serum INS level was detected by radioimmunoassay (RIA) according to the instructions of the kit.

### Measurements of biochemistry in liver tissue

Liver tissue homogenates were prepared by high-speed stirring of liver tissue in a 10-fold volume (v/w) of ice-cold PBS (for estimation of SOD and MDA) or anhydrous alcohol (for estimation of TC and TG), followed by 12000 rpm centrifugation at 4 °C for 15 min. The supernatant was then collected for subsequent analysis. Protein concentrations were assayed with a BCA method. Hepatic SOD, MDA, TC and TG were assayed using the same diagnostic kits as used for serum analysis.

Hepatic glycogen content was measured by the sulfuric anthrone methods^33^ according to the manufacturer’s instruction. Briefly, 1 g of frozen liver tissue was incubated with 30% KOH at 100 °C for 20 min, and diluted with ice-cold water. Suspension of the KOH digest were mixed slowly with the anthrone reagent (1:2, vol/vol) and boiled for 10 min for the development of color. The optical density was read spectrophotometrically within 2 h at 620 nm. The glycogen contents in liver tissue samples were expressed as mg/g liver protein.

### Histological assessment of liver

The liver tissues were resected and fixed in 10% formalin solution and then embedded in paraffin using a tissue-embedding procedure. After fixation, the tissue sections were cut into 4 μm sections and stained with H&E.

### Measurement of ROS in liver tissue

Superoxide (O(2)(-)) is one of ROS. To assess hepatic ROS production, sections of frozen rat liver tissue were prepared for DHE staining using a routine method. Photomicrographs were taken with a light microscope equipped with a camera (Olympus, Tokyo, Japan).

### Western blot analysis of protein expression in liver tissue

Equal amounts of protein from tissue homogenates were subjected to SDS-PAGE and transferred to PVDF membranes. The protein expression levels of SREBP-1c, FAS, ACCα, HMGCR, CYP7A1, IRS, p-IRS2 (Ser^307^), Akt, p-Akt (Ser^473^), GSK3β, p-GSK3β (Ser^9^) and CYP2E1 were detected with specific antibodies. Membranes were detected with primary antibodies and then probed with horseradish peroxidase-conjugated secondary antibodies using an ECL kit. β-actin was used as a loading control. The densities of immunoblot bands were performed with Quantity One software (Bio-Rad, Hercules, CA, USA).

### Statistical analysis

Data are expressed as the means ± SD. Data analysis was conducted with the IBMM SPSS 22.0 software package for Windows (SPSS Inc., Chicago, USA). Differences among the three groups were estimated via One-Way analysis of variance (ANOVA) followed by Tukey’s Post-Hoc test. P < 0.05 was considered statistically significant.

## Additional Information

**How to cite this article:** Ma, Z. *et al*. Beneficial effects of paeoniflorin on non-alcoholic fatty liver disease induced by high-fat diet in rats. *Sci. Rep.* **7**, 44819; doi: 10.1038/srep44819 (2017).

**Publisher's note:** Springer Nature remains neutral with regard to jurisdictional claims in published maps and institutional affiliations.

## Figures and Tables

**Figure 1:**
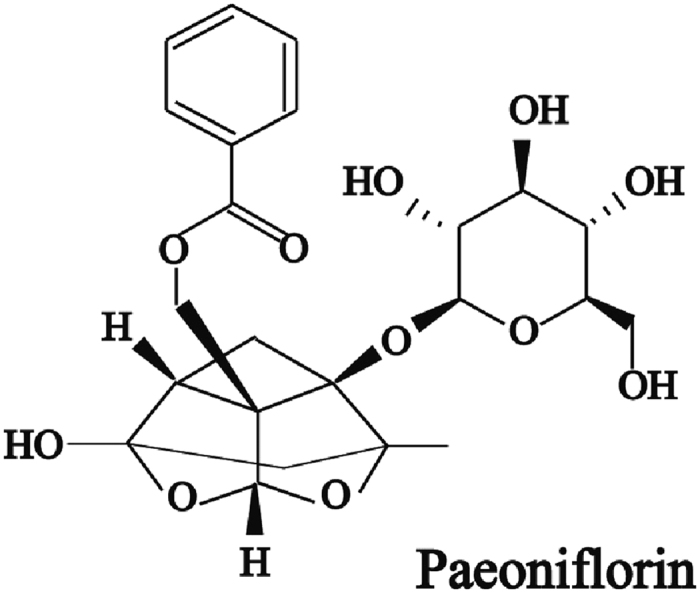
General structure of paeoniflorin.

**Table 1:** Effects of paeoniflorin on body weight and liver index.

Variables	Control	HFD	PF
Initial body weight (BW) (g)	150.1 ± 4.7	150.3 ± 5.9	149.6 ± 5.1
Final BW (g)	456.1 ± 11.8	496.5 ± 15.3*	481.6 ± 18.3
Liver index (100%)	2.59 ± 0.23	3.72 ± 0.10*	3.07 ± 0.14_#_

Control: normal control rats received a standard chow diet; HFD: high-fat diet-induced NAFLD rats received normal saline solution; PF: high-fat diet-induced NAFLD rats received paeoniflorin at a dose of 20 mg/kg/d; BW: body weight; Liver index calculated as liver weight/body weight × 100%. Data were expressed as the mean ± SD (n = 8 in each group). *P < 0.05 vs. Control group; ^#^P < 0.05 vs. HFD group.
